# Supplementary material for: Male DAT Val559 Mice Exhibit Compulsive Behavior under Devalued Reward Conditions Accompanied by Cellular and Pharmacological Changes
Source: Cells. 2022 Dec 15;11(24):4059. doi: 10.3390/cells11244059 (PMC9777203; doi:10.3390/cells11244059)
Supplement: Supplementary file 1 [file cells-11-04059-s001.zip › cells-2035438-supplementary.pdf]

Data Supplement:

Male DAT Val559 Mice Exhibit Compulsive Behavior Under Devalued Reward Conditions Accompanied by Cellular and Pharmacological Changes

Adele Stewart PhD[1,2,#], Gwynne L. Davis PhD[1,#], Lorena B. Areal PhD[1], Maximilian J. Rabil[1], Vuong Tran[1], Felix P. Mayer PhD[1], and Randy D. Blakely PhD[1,2,*]

[1]Department of Biomedical Science and [2]Brain Institute, Florida Atlantic University, Jupiter, FL.

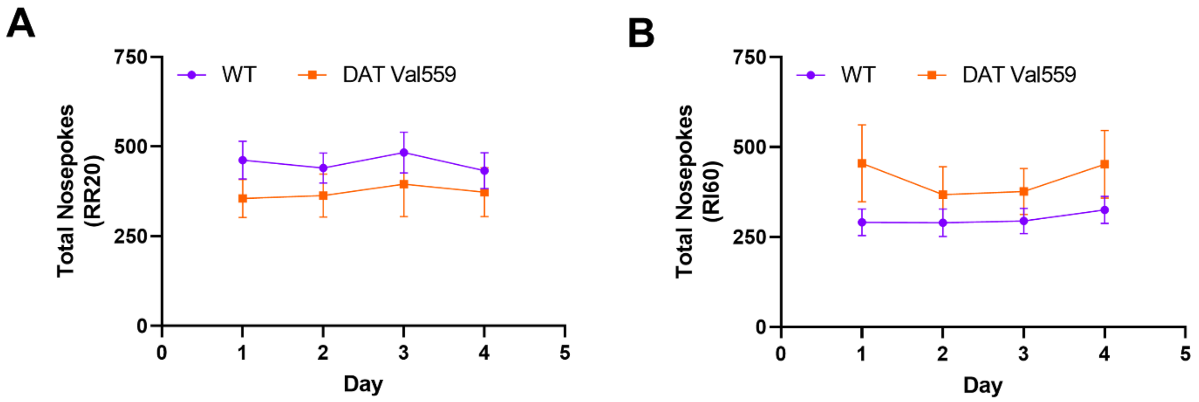

Figure S1. Instrumental responding is stable across the final days of training in the Goal vs Habit nose poke paradigm. WT (n=12) and DAT Val559 (n=10) males were subjected to a within subject lever pressing paradigm that utilizes contextual cues coupled to random ratio (RR) or random interval (RI) reinforcement schedules to bias animals toward goal-directed or habitual actions, respectively. Total nose pokes during the RR20/RI60 phase of the paradigm are depicted for the (A) RR and (B) RI context. There was no significant impact of genotype or day on total nose pokes for either context (See Table S2). Data were analyzed by two-way RM-ANOVA. Data are presented as mean ± SEM.

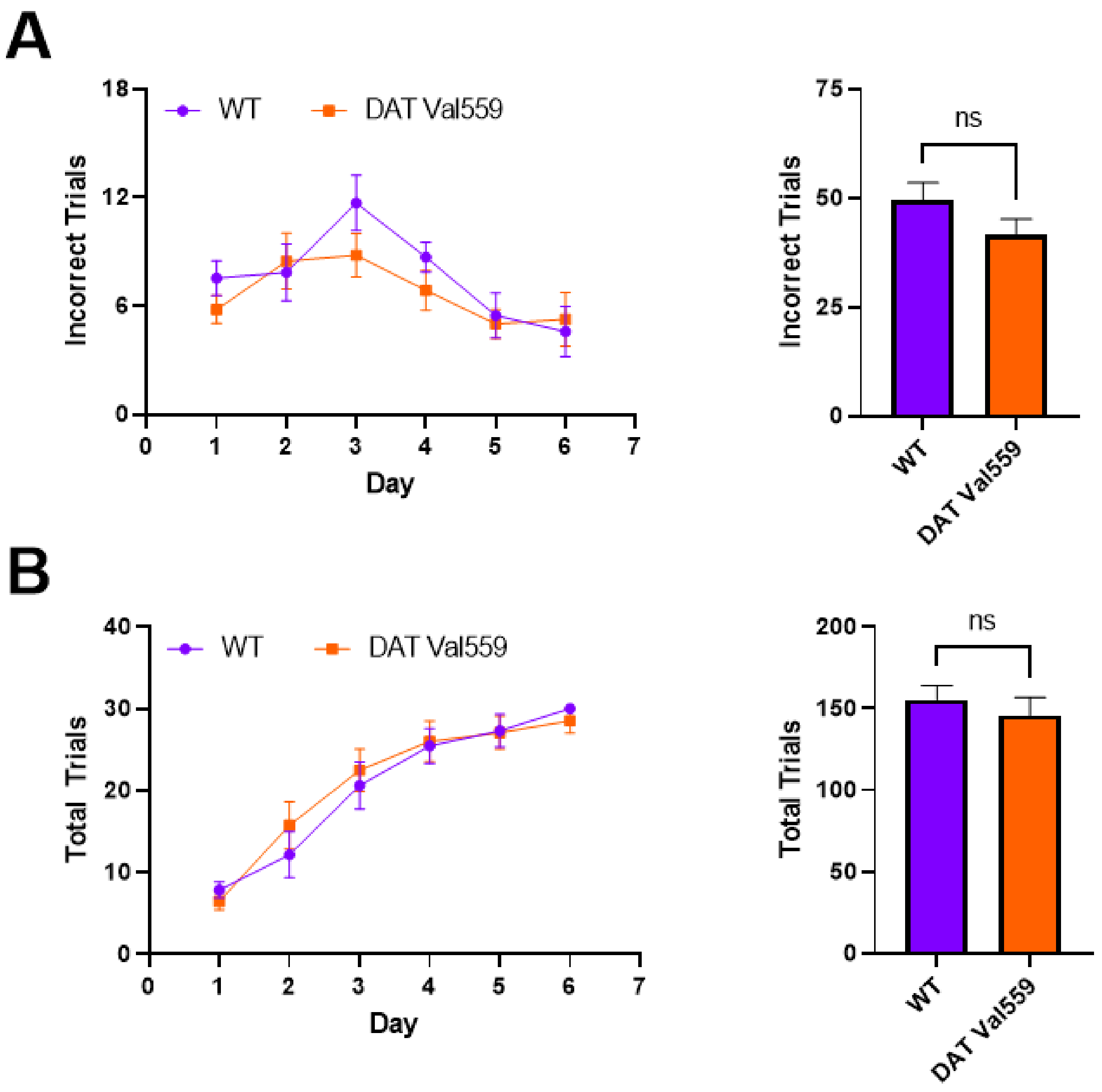

Figure S2. Supplemental reversal learning data. WT (n=14) and DAT Val559 (n=15) mice underwent pairwise discrimination training followed by a reversal phase. (A) Incorrect trials by day (left) and summed (right) during the reversal learning phase. (B) Trials completed by day (left) and summed (right) during the reversal learning phase. Data were analyzed by two-tailed student's t-test or two-way RM-ANOVA with Sidak's multiple comparison's test. ns = not significant. Data are presented as mean ± SEM.

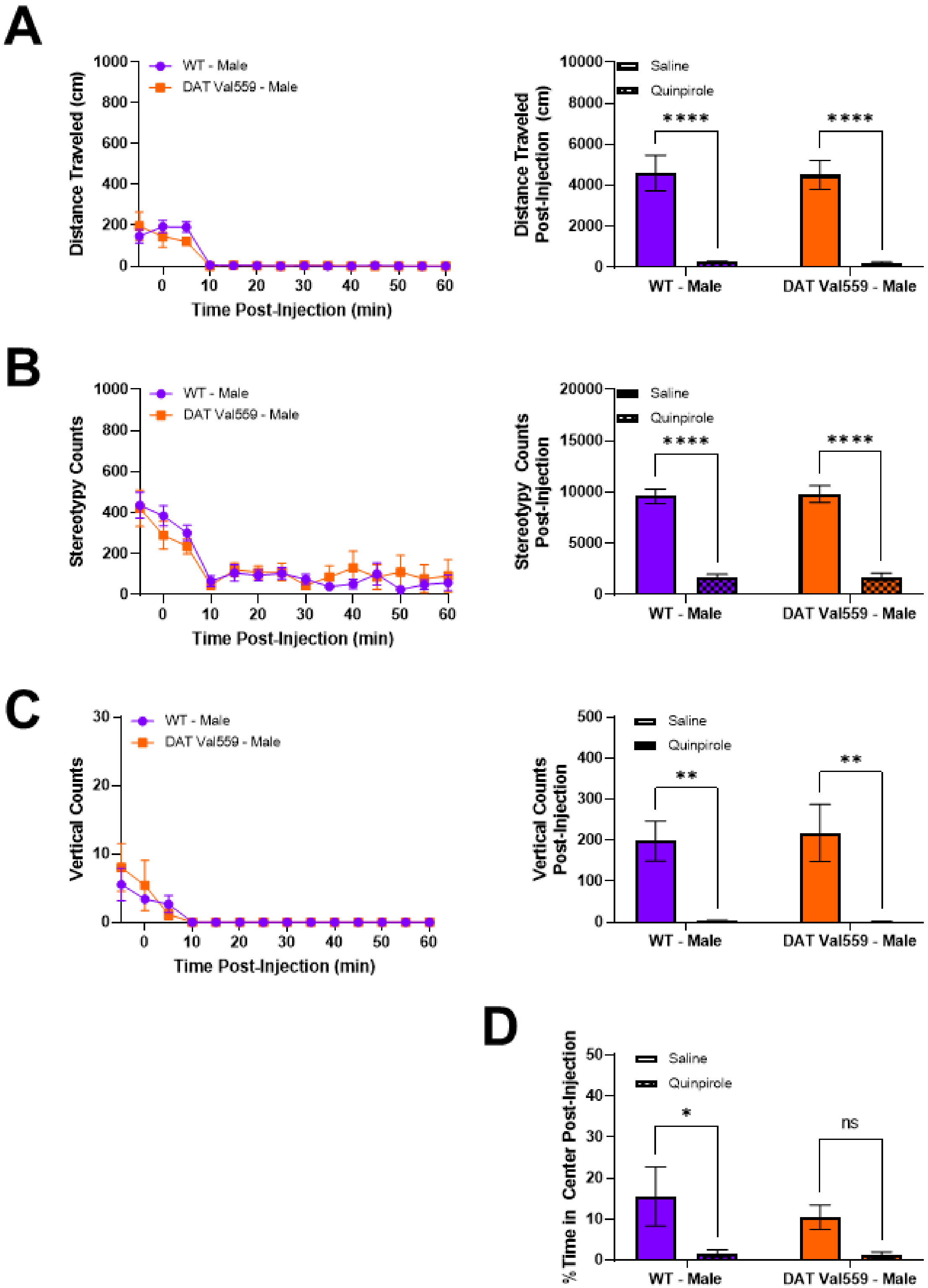
A
WT - Male
DAT Val559 - Male
Distance Traveled (cm)
Time Post-Injection (min)
Saline
Quinpirole
Distance Traveled Post-Injection (cm)
****
WT - Male
DAT Val559 - Male
B
Stereotypy Counts
Stereotypy Counts Post-Injection
C
Vertical Counts
Vertical Counts Post-Injection
**
D
% Time in Center Post-Injection
*
ns

Figure S3. Quinpirole-dependent locomotor suppression is comparable in WT and DAT Val559 mice. WT (n=8) and DAT Val559 (n=8) mice were given a single injection of the D2/D3 agonist quinpirole (1 mg/kg, i.p.) and locomotor activity recorded for 60 minutes post-injection. Datasets are presented in 5-minute time bins across the recording period and as summary data adding up all activity post-injection. (A) Horizontal distance traveled. (B) Stereotypic motor movements. (C) Vertical locomotor activity. (D) % Time spent in the center of the chamber. Data were analyzed by two-way ANOVA with Sidak's multiple comparison's test. **P* < 0.05, ***P* < 0.01, ****P* < 0.001, *****P* < 0.0001. ns = not significant. Data are presented as mean ± SEM.

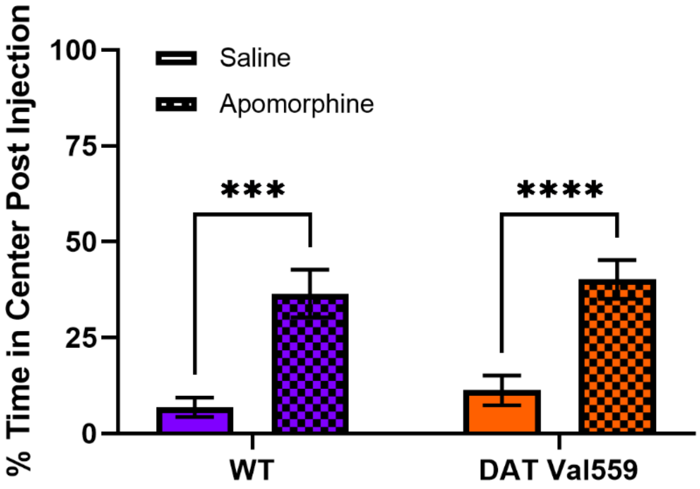

Figure S4. WT and DAT Val559 mice increase center occupancy in response to apomorphine. WT (n=13) and DAT Val559 (n=15) mice were given a single injection of the DA agonist apomorphine (5 mg/kg, s.c.) and locomotor activity recorded for 60 minutes post-injection. % Time spent in the center of the chamber after drug administration is depicted. Data were analyzed by two-way ANOVA with Sidak's multiple comparison's test. ***P < 0.001, ****P < 0.0001. Data are presented as mean ± SEM.

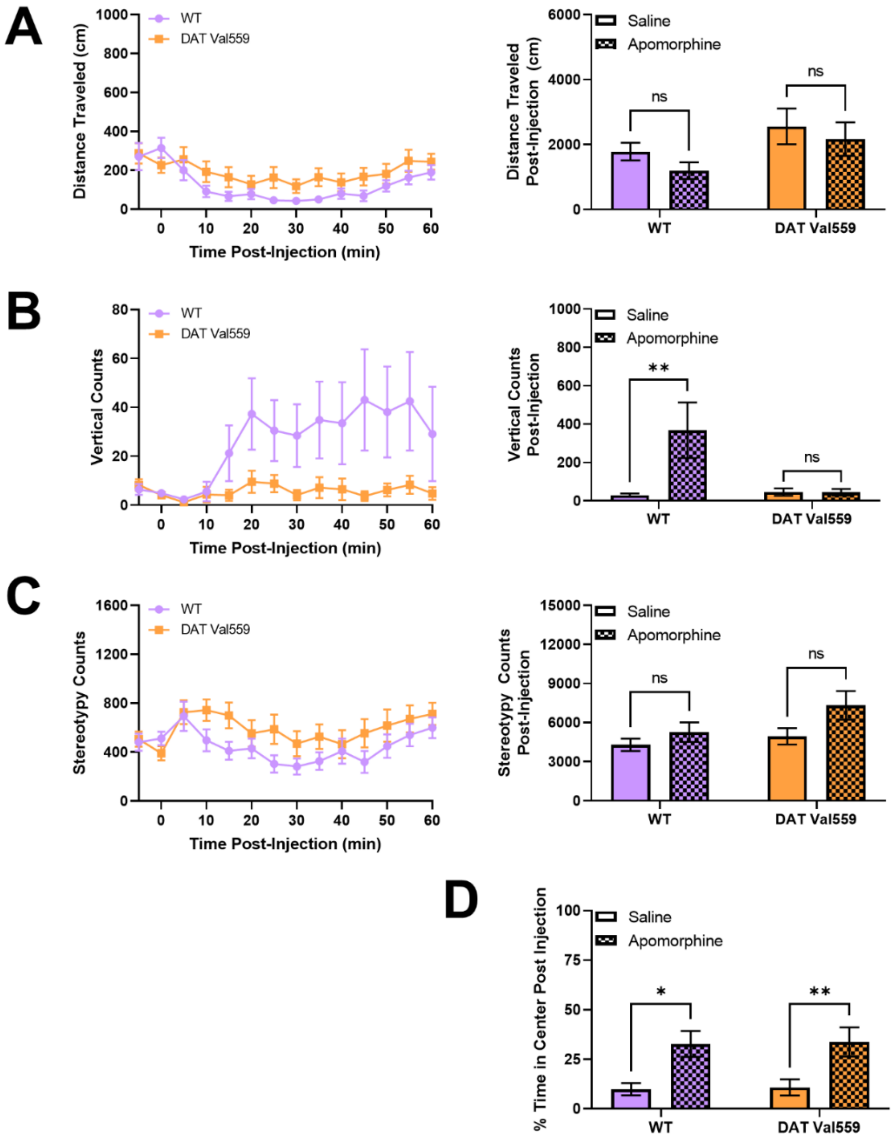

Figure S5. DAT Val559 females display a loss of apomorphine-dependent rearing. WT (n=14) and DAT Val559 (n=14) female mice were given a single injection of the DA agonist apomorphine (5 mg/kg, s.c.) and locomotor activity recorded for 60 minutes post-injection. Datasets are presented in 5-minute time bins across the recording period and as summary data adding up all activity post-injection. (A) Horizontal distance traveled. (B) Vertical locomotor activity. (C) Stereotypic motor movements. (D) Center occupancy. Data were analyzed by two-way ANOVA with Sidak's multiple comparison's test. *P < 0.05, **P < 0.01. ns = not significant. Data are presented as mean ± SEM.

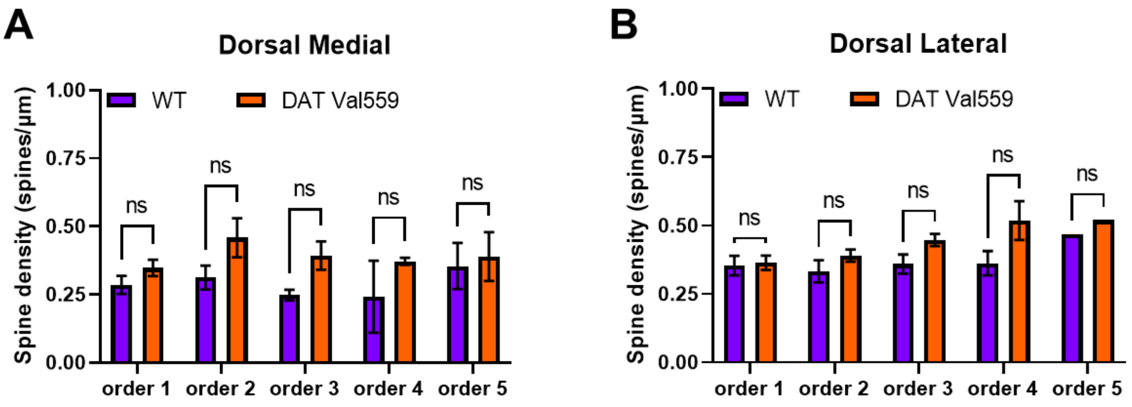

Figure S6. Supplemental dendritic spine data. Spine density in sections from WT (n=4) and DAT Val559 (n=4) mice was assessed utilizing Golgi staining coupled to brightfield microscopy. Spine densities were stratified based on degree of separation from the cell soma (order 1-5) in the (A) DMS and (B) DLS. Data were analyzed by two-way ANOVA with Sidak's multiple comparison's test. ns = not significant. Data are presented as mean ± SEM.

Table S1: Complete statistical analysis for data analyzed by Student's t-test. Cohen's *d* is provided as an indicator of effect size. For reference, *d* = 0.2 would be considered a 'small' effect size, 0.5 represents a 'medium' effect size and 0.8 a 'large' effect size

Figure	t, df	P value	Significant?	Effect Size, Cohen's *d*
2A	t=1.339, df=40	0.1881		0.412
2B	t=2.233, df=41	0.0311	*	0.676
2C	t=2.141, df=41	0.0383	*	0.653
2D	t=0.3684, df=41	0.7145		0.112
3C	t=2.045, df=27	0.0507		0.755
3D	t=2.419, df=27	0.0226	*	0.894
3E	t=1.904, df=25	0.0685		0.729
3F	t=0.7967, df=27	0.4326		0.295
5B	t=2.577, df=6	0.0419	*	1.821
5C	t=1.852, df=6	0.1135		1.310
S1A	t=1.473, df=26	0.1528		0.558
S1B	t=0.6684, df=26	0.5098		0.256

Table S2: Complete statistical analysis for data analyzed by two-way ANOVA. Eta squared (η^2) is provided as an indicator of effect size. For reference, η^2 = 0.01 indicates a small effect; η^2 = 0.06 indicates a medium effect; η^2 = 0.14 indicates a large effect.

Figure	Source of Variation	F (DFn, DFd)	P value	Significant?	Effect Size, η^2
1C	Interaction	F (3, 80) = 0.07582	P=0.9729		0.0028
	Genotype	F (1, 80) = 0.002305	P=0.9618		0.0000
	Context	F (3, 80) = 32.32	P<0.0001	****	0.5480
1D	Interaction	F (1, 37) = 0.1362	P=0.7142		0.0037
	Context	F (1, 37) = 1.271	P=0.2668		0.0332
	Genotype	F (1, 37) = 14.82	P=0.0005	***	0.2860
1E	Interaction	F (1, 40) = 1.549	P=0.2205		0.0373
	Food type	F (1, 40) = 245.2	P<0.0001	****	0.8598
	Genotype	F (1, 40) = 2.661	P=0.1107		0.0624
4A	Interaction	F (1, 52) = 0.1150	P=0.7359		0.0022
	Genotype	F (1, 52) = 1.656	P=0.2038		0.0309
	Drug	F (1, 52) = 0.1098	P=0.7417		0.0021
4B	Interaction	F (1, 52) = 0.1796	P=0.6734		0.0034
	Genotype	F (1, 52) = 3.799	P=0.0567		0.0681
	Drug	F (1, 52) = 1.371	P=0.2469		0.0257
4C	Interaction	F (1, 51) = 3.455	P=0.0688		0.0634
	Genotype	F (1, 51) = 7.226	P=0.0097	**	0.1241
	Drug	F (1, 51) = 16.10	P=0.0002	***	0.2400
5B	Interaction	F (2, 18) = 1.008	P=0.3847		0.1007
	Spine Type	F (2, 18) = 55.46	P<0.0001	****	0.8604
	Genotype	F (1, 18) = 6.901e-017	P>0.9999		0.0000
5C	Interaction	F (2, 18) = 1.041	P=0.3733		0.1037
	Spine Type	F (2, 18) = 62.42	P<0.0001	****	0.8740
	Genotype	F (1, 18) = 0.01895	P=0.8920		0.0011
S1A	Day x Genotype	F (3, 60) = 0.1960	P=0.8987		0.0097
	Day	F (2.515, 50.31) = 0.6441	P=0.5638		0.0312
	Genotype	F (1, 20) = 1.223	P=0.2819		0.1897
S1B	Day x Genotype	F (3, 60) = 0.6364	P=0.5945		0.0308
	Day	F (3, 60) = 1.316	P=0.2775		0.0617
	Genotype	F (1, 20) = 2.097	P=0.1631		0.2477
S3A	Interaction	F (1, 28) = 0.001124	P=0.9735		0.0000
	Genotype	F (1, 28) = 0.01867	P=0.8923		0.0007
	Drug	F (1, 28) = 58.76	P<0.0001	****	0.6773
S3B	Interaction	F (1, 28) = 0.03167	P=0.8600		0.0011
	Genotype	F (1, 28) = 0.03833	P=0.8462		0.0014

	Drug	F (1, 28) = 182.4	P<0.0001	****	0.8669
S3C	Interaction	F (1, 28) = 0.06037	P=0.8077		0.0022
	Genotype	F (1, 28) = 0.04678	P=0.8303		0.0017
	Drug	F (1, 28) = 23.33	P<0.0001	****	0.4545
S3D	Interaction	F (1, 27) = 0.3251	P=0.5733		0.0119
	Genotype	F (1, 27) = 0.4466	P=0.5096		0.0163
	Drug	F (1, 27) = 8.076	P=0.0084	**	0.2303
S4	Interaction	F (1, 52) = 0.006125	P=0.9379		0.0001
	Genotype	F (1, 52) = 0.7662	P=0.3854		0.0145
	Drug	F (1, 52) = 39.89	P<0.0001	****	0.4341
S5A	Interaction	F (1, 52) = 0.05409	P=0.8170		0.0010
	Genotype	F (1, 52) = 4.308	P=0.0429	*	0.0765
	Drug	F (1, 52) = 1.354	P=0.2499		0.0254
S5B	Interaction	F (1, 52) = 0.8340	P=0.3653		0.0158
	Genotype	F (1, 52) = 3.071	P=0.0856		0.0558
	Drug	F (1, 52) = 4.654	P=0.0356	*	0.0821
S5C	Interaction	F (1, 51) = 5.100	P=0.0282	*	0.0909
	Genotype	F (1, 51) = 4.162	P=0.0465	*	0.0754
	Drug	F (1, 51) = 5.019	P=0.0295	*	0.0896
S5D	Interaction	F (1, 52) = 0.0002238	P=0.9881		0.0000
	Genotype	F (1, 52) = 0.03106	P=0.8608		0.0006
	Drug	F (1, 52) = 17.19	P=0.0001	***	0.2484
S6A	Interaction	F (4, 20) = 0.3888	P=0.8141		0.0721
	Spine Type	F (4, 20) = 0.8154	P=0.5303		0.1402
	Genotype	F (1, 20) = 7.600	P=0.0122	*	0.2754
S6B	Interaction	F (4, 23) = 0.9695	P=0.4432		0.1442
	Spine Type	F (4, 23) = 2.519	P=0.0690		0.3045
	Genotype	F (1, 23) = 6.004	P=0.0223	*	0.2069
